# The genetic intractability of *Symbiodinium microadriaticum* to standard algal transformation methods

**DOI:** 10.1371/journal.pone.0211936

**Published:** 2019-02-19

**Authors:** Jit Ern Chen, Adrian C. Barbrook, Guoxin Cui, Christopher J. Howe, Manuel Aranda

**Affiliations:** 1 Red Sea Research Center, King Abdullah University of Science and Technology (KAUST), Thuwal, Saudi Arabia; 2 Department of Biological Sciences, Sunway University, Bandar Sunway, Malaysia; 3 Jeffrey Sachs Center on Sustainable Development, Sunway University, Bandar Sunway, Malaysia; 4 Department of Biochemistry, University of Cambridge, Cambridge, United Kingdom; Stony Brook University, UNITED STATES

## Abstract

Modern transformation and genome editing techniques have shown great success across a broad variety of organisms. However, no study of successfully applied genome editing has been reported in a dinoflagellate despite the first genetic transformation of *Symbiodinium* being published about 20 years ago. Using an array of different available transformation techniques, we attempted to transform *Symbiodinium microadriaticum* (CCMP2467), a dinoflagellate symbiont of reef-building corals, with the view to performing subsequent CRISPR-Cas9 mediated genome editing. Plasmid vectors designed for nuclear transformation containing the chloramphenicol resistance gene under the control of the CaMV p35S promoter as well as several putative endogenous promoters were used to test a variety of transformation techniques including biolistics, electroporation and agitation with silicon carbide whiskers. Chloroplast-targeted transformation was attempted using an engineered *Symbiodinium* chloroplast minicircle encoding a modified PsbA protein expected to confer atrazine resistance. We report that we have been unable to confer chloramphenicol or atrazine resistance on *Symbiodinium microadriaticum* strain CCMP2467.

## Introduction

Efforts to understand better the molecular mechanisms which govern the symbiosis between marine Cnidarians and their dinoflagellate symbionts have been hampered by the lack of genetically tractable model organisms. This is especially true for the symbiotic relationship between corals and dinoflagellates from the genus *Symbiodinium*. This interaction forms the bedrock of the coral ecosystem [1, 2] and yet is highly sensitive to relatively small changes in environmental conditions [3]. Abnormally high ocean temperatures have been identified as one of the key factors that can precipitate the breakdown of the *Symbiodinium*-coral symbiosis, which can lead to wide-spread, regional and even global coral bleaching events [4]. The predicted increase in ocean temperatures due to anthropogenic climate change is expected to accelerate this crisis [5].

One of the prevailing theories as to how coral reefs would be able to withstand rising ocean temperatures rests on the assumption that there are certain thermo-tolerant *Symbiodinium* strains which are able to form a more robust relationship with their host [6]. While the effectiveness of thermo-tolerant *Symbiodinium* strains in maintaining algal-coral symbiosis has been shown to be significant, the genetic basis of this robustness remains unknown. Without genetic tools and the absence of any viable method to carry out traditional genetic studies such as inbreeding and cross-breeding, isolating and confirming the identity of thermo-tolerance genes will be difficult.

Previous studies have described methods for transformation of free-living *Symbiodinium* cells. The first, published in 1997 by ten Lohuis and Miller describes the transformation of *Symbiodinium* CS-153 using silicon carbide whiskers [7]. In the ten Lohuis paper it was reported that the Cauliflower Mosaic Virus p35S and *Agrobacterium* nos and p1’2’ promoters were able to drive the expression of the reporter gene β-glucuronidase (GUS) and selectable markers (hygromycin and geneticin resistance genes) in *Symbiodinium*. Subsequently, two papers published in 2015 by Ortiz-Matamoros et al. described transformation of *Symbiodinium kawagutii*, *Symbiodinium* sp. Mf11.5b.1 and *Symbiodinium microadriaticum* MAC-CassKB8 using glass beads agitation with and without *Agrobacterium* [8, 9]. In these publications, the authors used the nos promoter to drive the expression of the *bar* gene which confers resistance to glufosinate, the active ingredient in the herbicide Basta (Bayer, Inc.). However, the authors of the paper note that their transiently transformed cells lost their chlorophyll and were unable to reproduce under herbicide selection.

We used previously published transformation protocols for *Symbiodinium* [7] as well as different standard protocols for algae based on electroporation, biolistics and agitation with glass beads to attempt to transform *S*. *microadriaticum* CCMP2467. Plasmid constructs utilizing the Cauliflower Mosaic Virus p35S promoter as well as putative endogenous *Symbiodinium* promoter and terminator regions identified from the S. *microadriaticum* genome [10] were used to drive the chloramphenicol resistance gene for nuclear transformation, while artificial *Symbiodinium* plastid minicircles modified to carry a mutated *psbA* gene that is predicted to confer resistance to the herbicide atrazine were used to carry out chloroplast-targeted transformation attempts. However, we were unable to obtain resistant strains under chloramphenicol or atrazine selection using either set of constructs.

We also carried out an attempted nuclear transformation using a construct carrying the geneticin resistance gene under the control of the CaMV p35S promoter and NosT terminator with *Symbiodinium* strain CS-153, the strain that was used in the ten Lohuis and Miller (1998) paper. After 10 weeks, we did not see any growth on geneticin agar plates or in geneticin liquid cultures.

## Results

### Antibiotic susceptibility test

Previous studies [7, 9] used notably high concentrations of hygromycin and geneticin (G418) of around 3 mg/ml in order to select for resistant *Symbiodinium* transformants, a process which required about three months as untransformed cells were able to survive up to eight weeks of antibiotics exposure.

**Fig 1:**
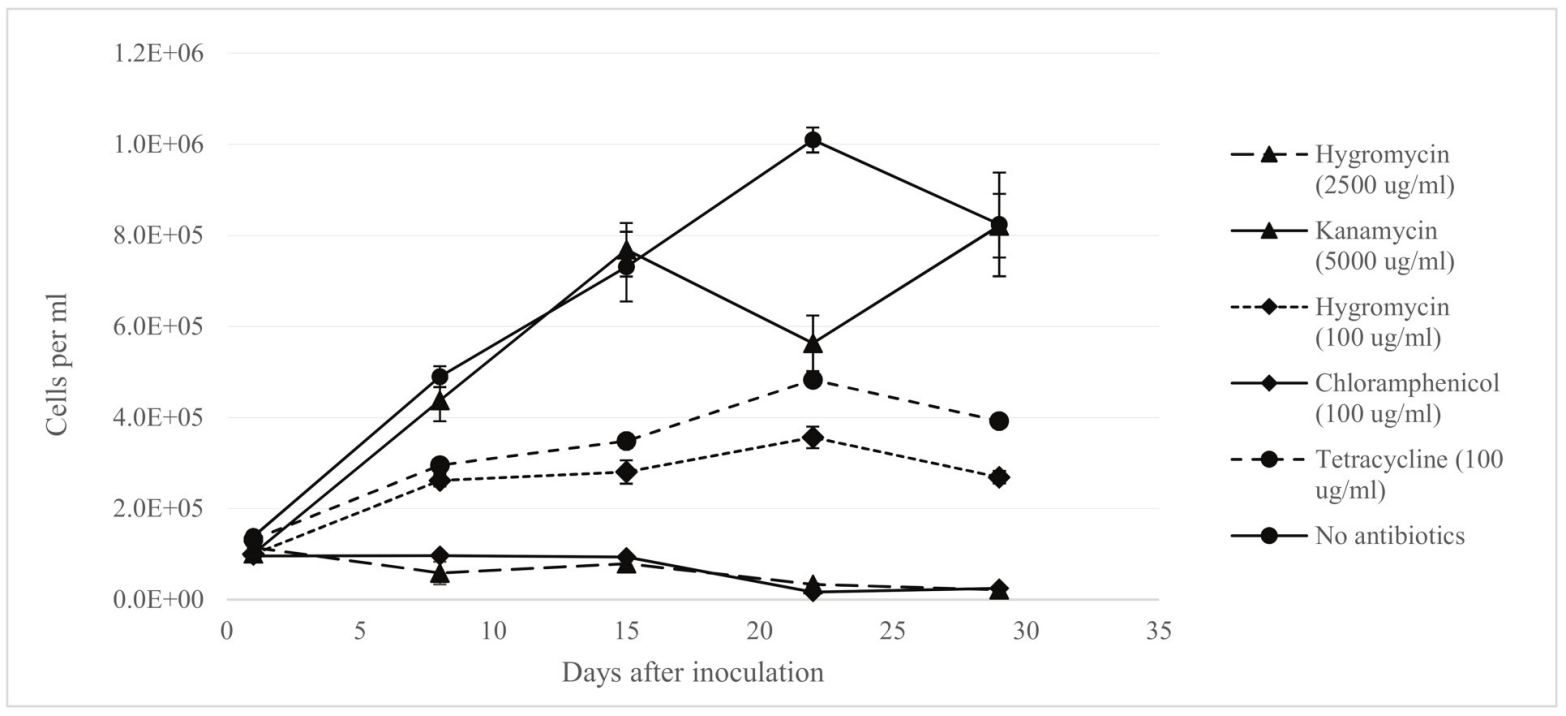
Growth of *Symbiodinium* CCMP2467 in f/2 liquid medium under antibiotic selection. Results of *Symbiodinium* growth were from three biological replicates, each measured four times using a FlowCAM. The error bars indicate standard error of the mean (S16 File).

In order to select a more cost-effective selection antibiotic and to confirm that our main transformation strain CCMP2467 had similar antibiotic tolerance levels to strain CS-153, we used liquid cultures to test the effectiveness of several antibiotics to determine a more potent and less expensive alternative to hygromycin and geneticin. We determined that 100 μg/ml of chloramphenicol was just as effective as 2.5 mg/ml of hygromycin in reducing the number of observed *Symbiodinium* CCMP2467 cells in culture (Fig 1). In addition, the cost of chloramphenicol is significantly lower than hygromycin or geneticin weight-for-weight across a range of suppliers (S1 Table). Therefore, we decided to use chloramphenicol instead of hygromycin or geneticin as our main antibiotic to select for transformants. A similar, independent test was also carried out to determine an effective selection concentration for atrazine resistance, which was found to be 150 ng/ml (S2 Table).

### Test for chloramphenicol resistance gene function

**Fig 2:**
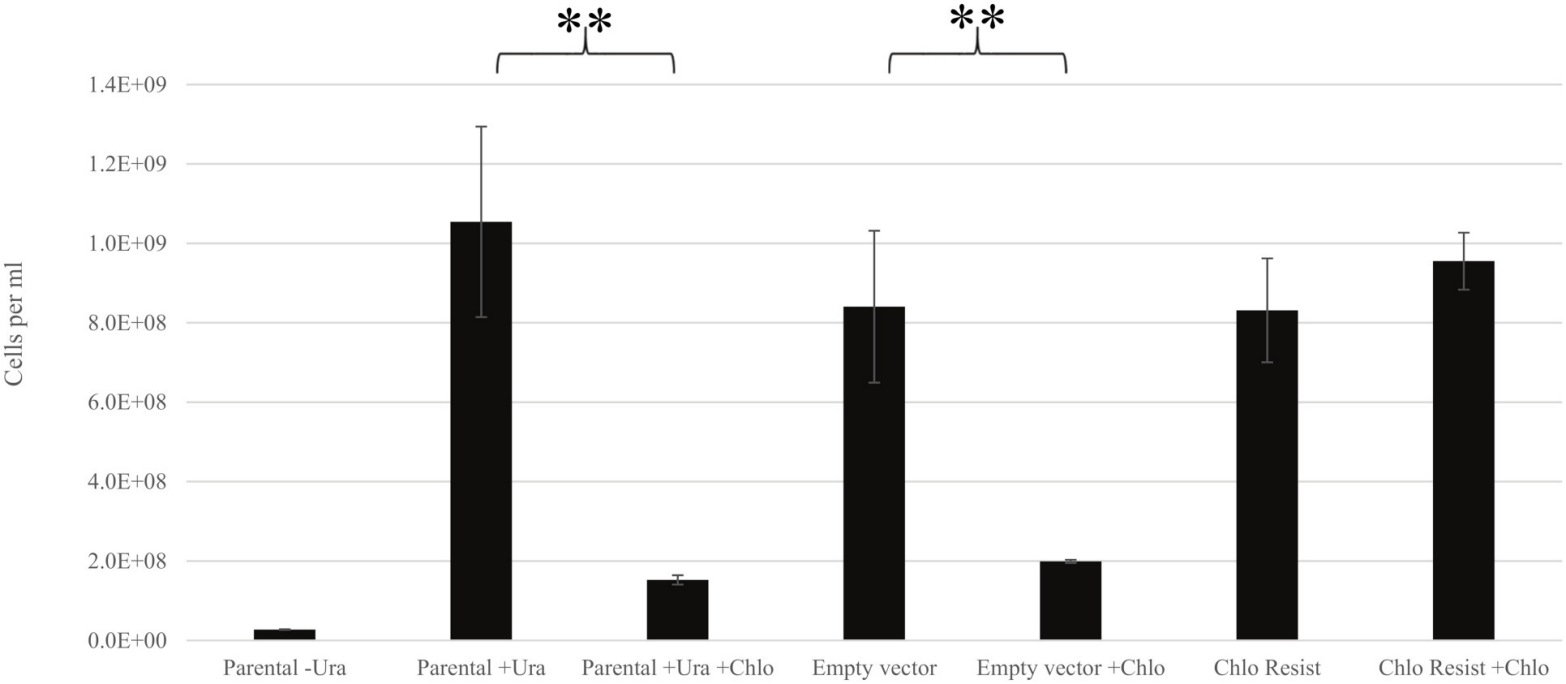
*S*. *cerevisiae* growth under chloramphenicol selection. “Parental” refers to the parental, untransformed *S*. *cerevisiae* yeast strain 31019b. “Empty vector” refers to the parental yeast strain transformed with a pYES2.1 vector carrying an eGFP coding region. “Chlo Resist” refers to the parental yeast strain transformed with a pYES2.1 vector carrying the chloramphenicol acyltransferase (CAT) resistance gene (ChloR). “+Chlo” indicates treatment of cultures with chloramphenicol. “+Ura” and “-URA” indicate the presence and absence of uracil supplement in the growth media respectively. Results are from five biological replicates. Asterisks indicate significantly different means with a p-value of < 0.01 using Student’s t-test with unequal variance. T-test was carried out with a significance value of 0.05. Error bars indicate standard error of the mean (S17 File).

We tested the functionality of the chloramphenicol resistance gene using *Saccharomyces cerevisiae* expression vectors in *S*. *cerevisiae* (Fig 2). Under galactose induction, yeast cultures were grown with and without chloramphenicol selection (4 mg/ml) to show that the presence of the chloramphenicol resistance gene (ChloR) under the control of the *GAL1* promoter was necessary and sufficient to confer increase chloramphenicol resistance to yeast cells. The minimal growth medium used for this experiment was supplemented with 3% glycerol and 0.5% galactose in order to force yeast cells to grow aerobically while providing a low level of galactose to induce *GAL1* promoter expression. The results showed that yeast cells transformed with the pYESChloR construct (S1 File), which contains the ChloR gene under the control of the *GAL1* promoter, became more resistant to chloramphenicol compared to the control untransformed strain (31019b) and the strain transformed with the empty vector pYESeGFP (S2 File). We present this as evidence for the ability of the *ChloR* gene to facilitate chloramphenicol resistance when expressed as a transgene.

### Promoter region identification

**Fig 3:**
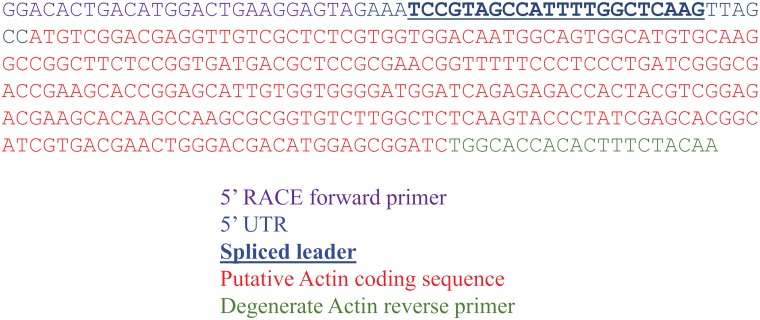
5’ RACE results of actin transcript to identify 5’ UTR sequence.

In order to identify suitable promoter regions we chose a set of five expressed genes including those for actin, β-tubulin A, β-tubulin B, Hsp90, and PsbJ from the *S*. *microadriaticum* genome as confirmed by transcriptomic data (S3 Table) from Chen et al. [11]. To identify the correct start codon for the actin and PsbJ genes we performed 5’ RACE on transcripts to sequence their 5’ UTRs. For the actin gene, the 5’ RACE results confirmed the presence of a spliced leader 55 bp upstream of the first ATG site (Fig 3). This particular spliced leader sequence is notable for being the most common one in the *Symbiodinium kawagutii* transcriptome, being found in 6235 out of 6501 full-length *S*. *kawagutii* cDNAs containing a complete spliced leader sequence [12]. Using the coding sequence, we did a BLAST search on the *S*. *microadriaticum* genome [10] and identified gene model Smic17360 as the best hit sequence for our 5’ RACE result. We then amplified an approximately 2 kb region upstream from the putative start ATG to serve as the promoter element for the pAct-ChloR-ActT construct. The 3’ terminator sequence used for this construct was the 500 bp region downstream of the stop codon of Smic17360.

**Fig 4:**
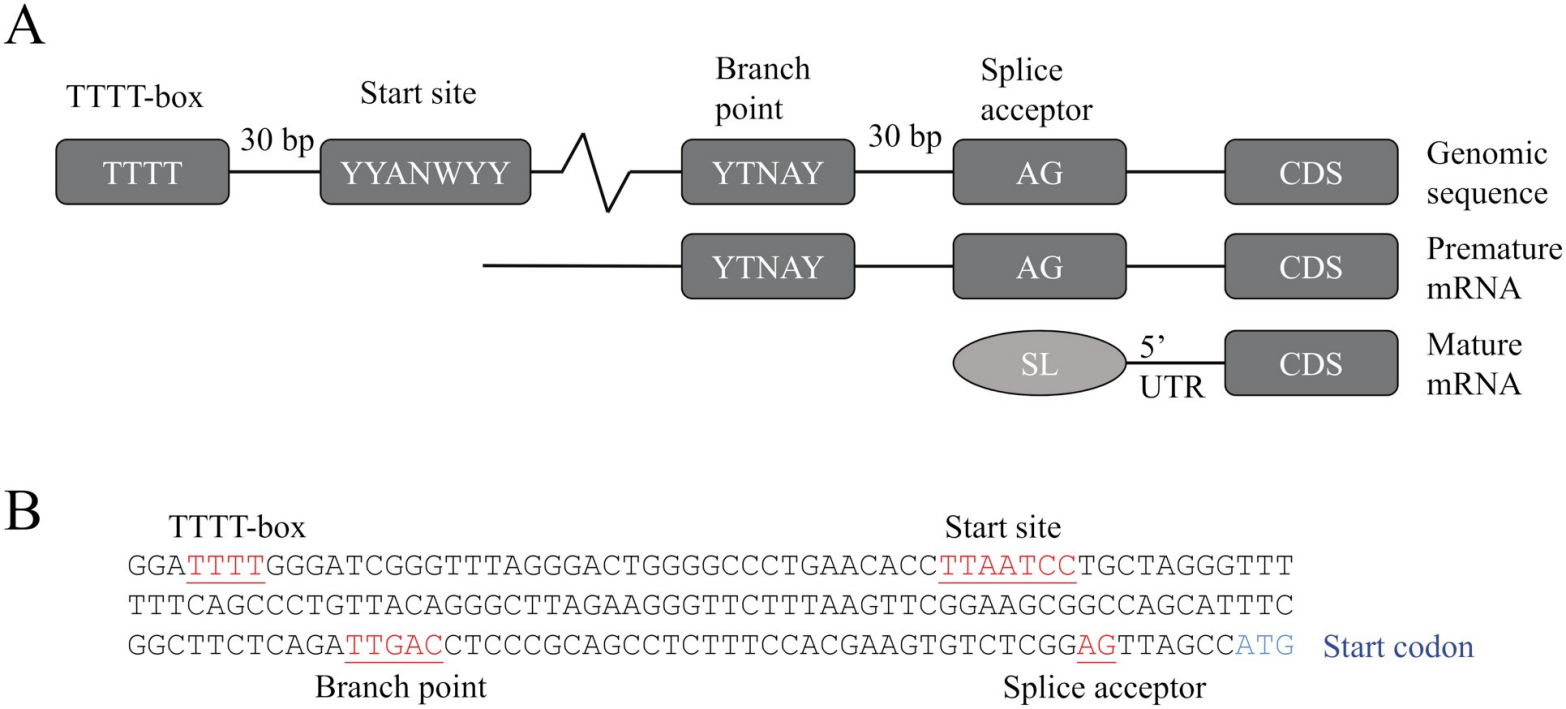
Unique promoter architecture in *Symbiodinium*. (A) A schematic view of the proposed promoter (TTTT-box) relative to the putative transcription start site, splice branch point and acceptor site, upstream of the coding region (CDS) in the genomic sequence of *S*. *kawagutii* genes. For comparison, premature mRNA and mature mRNA are also shown. Figure was adapted from Lin et al. (2015). (B) Genomic sequence of *S*. *microadriaticum pAct* promoter 175 bp upstream of the putative start codon, with promoter elements as described in (A).

Within the 2 kb promoter region upstream of Smic17360 (encoding actin), we identified all elements that have been noted as important for transcript expression and leader sequence *trans*-splicing (Fig 4A) [13]. A TTTT-box motif 34 bp sequence upstream of a potential transcriptional start site (YYANWYY) can be found as well as a branch point (YTNAY) present 32 bp upstream of the splice acceptor (AG) sequence (Fig 4B).

We carried out a similar 5’ RACE validation experiment to define the *PsbJ* promoter (*pPsbJ*) region (S1 Fig). In most photosynthetic organisms, the *psbJ* gene is located in the chloroplast genome as part of the *psbEFLJ* operon. However, in the *S*. *microadriaticum* genome the *PsbJ* gene exists as a three copy tandem repeat in the nucleus (S1 Fig), which lends support to previous reports that PsbJ is not encoded on any of the *Symbiodinium* chloroplast minicircles [14].

For the nuclear constructs based on the β-tubulin A, β-tubulin B and Hsp90 promoters, we took the 3 kb region upstream of the predicted start codon as the putative promoter region and a 400 bp 3’ region originating downstream of the endogenous Hsp90 stop codon as a terminator region (S3–S5 Files). These regions were selected only based on *in silico* analysis, and were not independently identified via RACE.

### Transformation construct design

**Fig 5:**
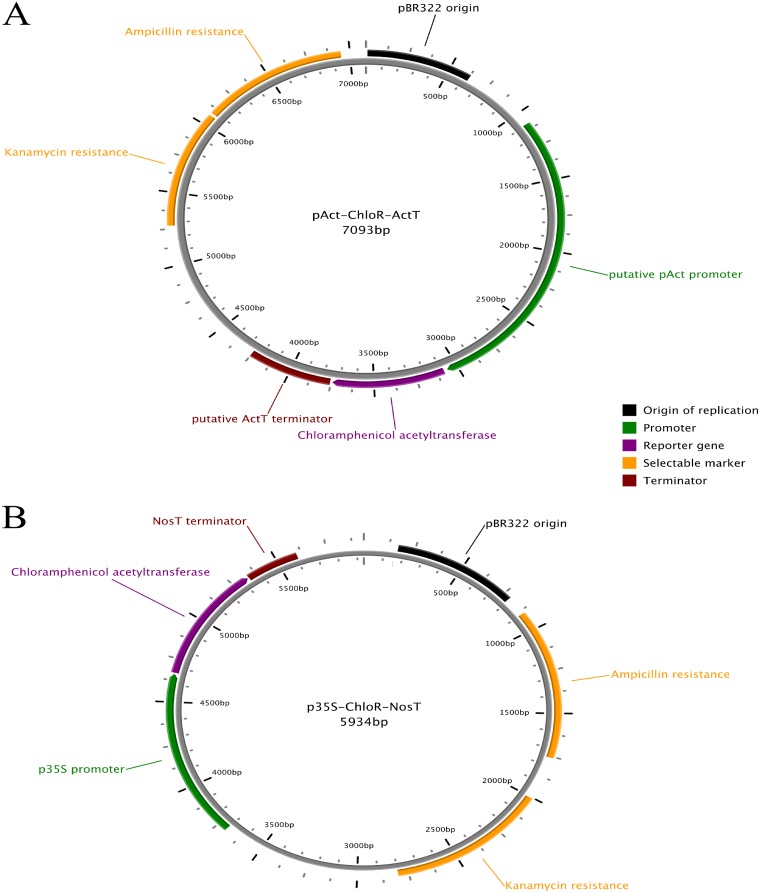
Vector maps for selected transformation plasmids. A) Vector map for plasmid pAct-ChloR-ActT, constructed using the pCR2.1 vector backbone. B) Vector map for plasmid p35S-ChloR-NosT, constructed using the pCR4 vector backbone.

Plasmids were constructed to carry the chloramphenicol (*ChloR*), geneticin (*GenR*) or the atrazine (*psbA*^*S264G*^) resistance gene under the control of a series of different gene promoters and terminators. An example of this is plasmid pAct-ChloR-ActT, which contains a putative actin promoter sequence and a putative actin terminator sequence flanking the *ChloR* gene (Fig 5A). We use the term *ChloR* here to describe the chloramphenicol acetyltransferase (*CAT*) gene which confers resistance to chloramphenicol and *GenR* to describe the neomycin phosphotransferase II (*nptII*) gene that confers resistance to geneticin G148. Similarly, the gene we term *psbA*^*S264G*^ is a variant of the *psbA* gene that results in an amino acid substitution that is predicted to confer resistance to atrazine.

The plasmids constructed can be classed into two major groups based on the target site of plasmid transformation (Table 1). Chloramphenicol and geneticin constructs, derived from commercial *E*. *coli* vector backbones, were designed for nuclear *Symbiodinium* transformation while atrazine constructs were designed as artificial chloroplast minicircle chromosomes. In addition to the promoter and terminator regions previously described, the p35S promoter and nos terminator were also used to construct the p35S-ChloR-NosT (Fig 5B) and p35S-Neo-2A-eCFP constructs, as it has been reported previously that these expression elements were sufficient to drive the expression of the hygromycin resistance gene in *Symbiodinium*.

**Table 1:** List of constructs.

Construct name	Plasmid backbone	Promoter origin	Resistance gene	Terminator origin
p35S-ChloR-NosT	pCR4	Cauliflower mosaic virus 35S	Chloramphenicol acetyltransferase	Agrobacterium Nopaline synthase
pAct-ChloR-ActT	pCR2.1	Symbiodinium SmicGene17360	Chloramphenicol acetyltransferase	Symbiodinium SmicGene17360
pBTubA-ChloR	pCR2.1	Symbiodinium SmicGene25527	Chloramphenicol acetyltransferase	(no terminator)
pBTubB-ChloR	pCR2.1	Symbiodinium SmicGene4192	Chloramphenicol acetyltransferase	(no terminator)
pHsp90-ChloR-Hsp90T	pCR2.1	Symbiodinium SmicGene33536	Chloramphenicol acetyltransferase	Symbiodinium SmicGene33536
pPsbJ-ChloR	pCR2.1	Symbiodinium SmicGene31399	Chloramphenicol acetyltransferase	(no terminator)
pPsbA^S264G^GEM	Chloroplast minicircle	Endogenous PsbA	Mutant PsbA, resistant to atrazine	Endogenous PsbA
pPsbAGEM	Chloroplast minicircle	Endogenous PsbA	none	Endogenous PsbA

Detailed plasmids maps for all constructs in this table can be found in the S6–S13 Files.

The artificial chloroplast minicircle constructs are fusions of a PCR-amplified *psbA* minicircle with the cloning vector pGEM-T-Easy, creating a simple shuttle vector for use in transformation experiments (pPsbAGEM). The sequence details of the shuttle vector can be found in S13 File. The *psbA* minicircle of *S*. *microadriaticum* was amplified with back-to-back primers. The primers were designed to anneal to regions of DNA just downstream of the *psbA* coding region, since subsequent inclusion of the vector sequence here seemed less likely to disrupt promoter or origin of replication sequences. The shuttle vector pPsbAGEM was then modified by site-directed mutagenesis to create pPsbA^S264G^GEM which contains two nucleotide changes that change the serine codon for residue 264 to a glycine codon and has a further synonymous base change in a neighbouring codon to act as a marker.

### Transformation details (*S*. *microadriaticum* strain CCMP2467)

A brief summary of the various transformation experiments carried out can be seen in Table 2, and the details of each transformation can be found in S4 Table. 45 sets of transformation experiments were carried out using strain CCMP2467 for a total of 207 transformation samples, including control samples (i.e. no plasmid control). Various transformation conditions for each transformation protocol were tested, and all treated cultures were kept under observation for at least 3 months in liquid culture with some of the experiments also selected for on agar plates.

**Table 2:** Summary list of the number of experiments carried out grouped by transformation method.

Transformation method	Number of experiments	Number of samples
Electroporation	17	69
Biolistics	9	57
Glass beads agitation	9	21
Silicon carbide whiskers agitation	7	31
FuGENE transfection	3	29
**Total**	**45**	**207**

Details on each transformation can be found in S4 Table.

Electroporation was the most used transformation method both in terms of the number of experiments and the number of samples treated. Both exponential decay and multi-square wave pulses were used, with field strengths ranging from 20 V to 2.2 kV. For biolistics, both gold and tungsten particles of various sizes were used with varying rupture disks strengths ranging from 900 to 1550 psi. The least tested method, FuGENE transfection, was only tested three times, using the recommended manufacturer’s protocol, with the variable factors being the amount of plasmid DNA used and the ratio of DNA to FuGENE solution (S4 Table).

In both solid and liquid tests, transformations were considered to have failed when cells in the no-antibiotic/no-herbicide control liquid cultures/agar plates became visibly decoloured due to senescence, while at the same time no growth could be observed in the selection cultures/plates. For agar plates, control culture senescence usually took around six to eight weeks and for liquid cultures it was around three months.

For the selective agar plates, none of the cultures tested survived more than four weeks after attempted transformation. For liquid culture selection, any cultures which showed signs of growth after roughly one month were sub-cultured into fresh f/2 liquid or agar with the appropriate selection antibiotic/herbicide. Although we did observe that some of these cultures began to grow under selection after several months in liquid culture, none of these treated cultures was able to grow after sub-culturing in fresh selection medium (data not shown). We therefore considered these growths as the result of non-transformed cells that were simply able to outlast the effective period of their respective selection antibiotic/herbicide.

### Transformation details (*Symbiodinium* strain CS-153)

We concentrated most of our transformation efforts on the *Symbiodinium* strain CCMP2467 because the genome of this strain had been sequenced and cultures of this strain are currently being used for other research projects in our group. However, the published protocol by ten Lohuis and Miller [7] used a different *Symbiodinium* strain, known as CS-153, isolated from a different cnidarian and geographic location from CCMP2467 (see Materials and methods). In addition, our nuclear transformation constructs were designed to use chloramphenicol rather than hygromycin or geneticin as selection markers.

We therefore decided to replicate as closely as possible the materials and methods used previously (ten Lohuis and Miller (7); David Miller, personal communication). We carried out a silicon carbide whiskers agitation transformation using *Symbiodinium* strain CS-153 with our chloramphenicol and geneticin constructs (Table 3) as the original ten Lohuis and Miller constructs were no longer available. The two geneticin constructs used contained the neomycin/geneticin resistance gene, Transposon Tn5 Aminoglycoside 3’ phosphotransferase (GenR), under the control of a *Chlamydomonas reinhardtii* chimeric HSP70A/RBCS2 promoter (S14 File) and a p35S promoter (S15 File).

**Table 3:** List of constructs used for CS-153 transformation using silicon carbide whiskers.

Construct name	Plasmid backbone	Promoter origin	Resistance gene	Terminator origin
p35S-ChloR-NosT	pCR4	Cauliflower mosaic virus 35S	Chloramphenicol acetyltransferase	Agrobacterium Nopaline synthase
pAct-ChloR-ActT	pCR2.1	Symbiodinium SmicGene17360	Chloramphenicol acetyltransferase	Symbiodinium SmicGene17360
pPsbJ-ChloR	pCR2.1	Symbiodinium SmicGene31399	Chloramphenicol acetyltransferase	(no terminator)
pChlamy3-GenR-GAmCherry	pChlamy_3	Chimeric Chlamydomonas HSP70A/RBCS2	Transposase Tn5 Aminoglycoside 3'-phosphotransferase	(no terminator)
p35S-GenR-eCFP-NosT	pChlamy_3	Cauliflower mosaic virus 35S	Transposase Tn5 Aminoglycoside 3'-phosphotransferase	Agrobacterium Nopaline synthase

Plasmid maps can be found in the S6, S7, S9, S14 and S15 Files.

Using the ten Lohuis and Miller [7] silicon carbide whiskers agitation protocol, two-week old *Symbiodinium* cultures at a cell density of roughly 3 × 10^5^ cells/ml were harvested and transformed with five different constructs (Table 3) and a “no plasmid” control. CS-153 cells after transformation attempts were then selected in liquid cultures and on top agar plates with the appropriate antibiotics. In the case of geneticin selection, we used the same concentration (3 mg/ml) that ten Lohuis and Miller [7] used. We did not detect any significant growth in liquid media or visibly growing colonies on selection plates even after 15 weeks of observation.

## Discussion

Our attempts to transform *S*. *microadriaticum* CCMP2467 stably have not been successful despite the use of a wide variety of constructs and standard transformation methods known to work successfully in the transformation of other microalgae. We have tested both transgenic (p35S) and endogenous promoters (pAct, pBTubA, pBTubB and pHsp90) to drive ChloR expression in the cytoplasm as well as an endogenous promoter from the gene for a nuclear-encoded protein targeted to the chloroplasts (pPsbJ). We have also attempted to introduce into *Symbiodinium* chloroplasts artificial minicircles that contain a modified *psbA* gene that is potentially able to confer resistance to atrazine.

In terms of transformation methods, we have tried to transform *Symbiodinium* using standard algal transformation techniques such as silicon carbide whisker agitation, biolistics (particle bombardment), electroporation and glass bead agitation. In addition, we used FuGENE transfection media to see if methods more commonly used to transfect animal cells were effective. We did not test *Agrobacterium*-mediated transformation, as Ortiz-Matamoros, Islas-Flores [8] reported that this method was able to create only transient, rather than stable, transformants that were unable to replicate or maintain normal pigmentation under Basta herbicide selection.

We also tested if the *Symbiodinium* strain CS-153 was more genetically tractable to the silicon carbide whiskers agitation transformation protocol published by ten Lohuis and Miller [7]. Although both CS-153 and CCMP2467 are classified as clade A *Symbiodinium* strains, we do not know the actual genetic distance between the two strains, and they may therefore actually be more akin to separate algal species than strains of the same species. Unfortunately, we did not observe any transformants with CS-153 either, in liquid culture or agar plates. We do acknowledge, however, that this attempt was only carried out once, and further optimization may be required for successful transformation of CS-153 using silicon carbide whiskers agitation.

At this point, we would like to present some speculations on our part about why we have been unable to carry out the nuclear transformation of *Symbiodinium*. We propose that the primary issue has to do with the physiology of the *Symbiodinium* nuclei itself. *Symbiodinium*, like all core dinoflagellates, possesses a unique nuclear arrangement, known as the dinokaryon. For example, one major feature of dinokaryon nuclei is that the chromosomes of the dinokaryon are permanently condensed throughout the cell lifecycle and exist in a liquid crystalline state [15]. Another example is the near lack of classical histones associated with the chromatin structure of the condensed *Symbiodinium* chromosomes, and the extreme 10:1 ratio of DNA to protein as compared to 1:1 in other eukaryotes [15]. Historically, these dinokaryon-specific features were deemed unique enough to raise the possibility that dinoflagellates were a sister group all other eukaryotes [16]. Taken together, there is a strong possibility that the structural and physiological characteristics of the dinokaryon might be different enough from the nuclei of “normal” eukaryotes that we may be unable to rely on the assumption that transgenic DNA will be integrated into the *Symbiodinium* genome as usually happens with other eukaryotes. In other words, we believe that the methods of DNA delivery into the *Symbiodinium* cells themselves are working but not the genome integration step, as evidenced by Nimmo et al. [17] who report that chloroplast-targeted transformation of dinoflagellates can be achieved. Dinoflagellate chloroplasts genomes are not thought to be significantly physiologically different from other “normal” chloroplast genomes and transformations using “origin-of-replication” plasmids/artificial chloroplast chromosomes avoid the need for genomic integration. In fact, since the dinoflagellate chloroplast genome is actually fragmented into “mini-circles”, each containing one ORF, rather than the canonical single chloroplast genome, it is arguably easier for whole chloroplast mini-circle replacement transformation to take place compared to regular chloroplasts plasmid replacement or integration. Note that we have indeed tried transforming *Symbiodinium* with constructs targeted to the chloroplasts and although the conditions that we used did not yield successfully transformed cells, we believe that there is merit in optimizing the protocol for *Symbiodinium* chloroplast transformation (as opposed to nuclear transformation) using standard transformation methods, particularly given that chloroplast transformation of the free-living dinoflagellate *Amphidinium carterae* has now been reported [17].

Another dinokaryon-specific trait that is not fully understood is the presence of high levels of the modified base 5-hydroxymethyluracil (5-hmU), which is a substitute for thymine (T). In several *Symbiodinium* species, the percentage of T substituted by 5-hmU in the genome is around 40%, with *S*. *microadriaticum* reported as having 48.1% [18]. This modified base may play a significant role in self-recognition or gene expression similar to CpG methylation [19, 20], and our current inability to replicate or mimic this phenomenon may be affecting the stability and the ability of our transgenic constructs to be retained, integrated and expressed in the *Symbiodinium* genome. Although enzymes that could be used to modify plasmid DNA *in vitro* are commercially available, specifically the Tet enzyme, the conversion rate of T to 5-hmU is far too low, being in the range of 1 conversion for every 250 T residues [21], as opposed to substitution of around 40% of T in *Symbiodinium* genomes. Furthermore, it has been reported that the distribution of 5-hmU in Dinoflagellate genomes is not uniformly interspersed throughout the genome [22], implying that random *in vitro* conversion of T to 5-hmU in transgenic constructs will not contain site-specific epigenetic information and may not solve the problem of self-recognition or gene expression in the context of the *Symbiodinium* dinokaryon.

With regards to *Symbiodinium* nuclear transformation specifically, we therefore strongly recommend that efforts must first be made to either identify a confirmed mechanism for DNA integration in the genome of dinoflagellates in general and *Symbiodinium* in particular, for example Cre-Lox-like mediated recombination or retrotransposon-based integration, or find a method to construct episome-type plasmids which can replicate independently of nuclear chromosomes. These mechanisms can then be harnessed to facilitate the stable transformation of transgenes into the dinokaryon of *Symbiodinium* for long-term genetic and molecular research. In addition, there have been recent publications that describe techniques that can be used to sequence modified base pairs, specifically 5-hmU [23, 24]. Sequencing the 5-hmU epigenome of *S*. *microadriaticum* will hopefully reveal the epigenetic pattern of 5-hmU and might provide some clues as to whether these patterns are involved in self-recognition and how best to overcome this problem.

In conclusion, our results illustrate the resistance of *Symbiodinium*, particularly *Symbiodinium microadriaticum* CCMP2467, to well-established algal transformation methods. Since the first reported publication by ten Lohuis and Miller [7], to our knowledge, the only other primary papers concerning *Symbiodinium* transformations are those of Ortiz-Matamoros et al. (8, 9). Ortiz-Matamoros et al. reported transient expression of GFP in *Symbiodinium* using glass bead transformations and *Agrobacterium tumefaciens*-assisted glass bead transformations. However, these methods were associated with loss of photosynthetic pigments and inability of cells to replicate under Basta herbicide selection. Taken together, these results strongly suggest that this particular algal group may require novel methods and techniques for successful transformation and editing of the genome.

## Materials and methods

### Yeast transformation and chloramphenicol resistance test

Yeast strain 31019b was kindly provided to us by Prof. Brent N. Kaiser from the University of Sydney. The S.c. EasyComp Transformation Kit (#K505001) (Thermo Fisher Scientific, Waltham, Massachusetts, US) was used to make transformation competent 31019b cells. The following primers were used to PCR amplify eGFP and ChloR coding region fragments.

eGFP F1 (5’-TCGACCTGCAGGCGGCCGCAC-3’)
eGFP R1 (5’-CTTCTTGTCCATGGTGAAGGGG-3’)
ChloR F1 (5’-ATGGAGAAAAAAATCACTGGATATACCACCGTTGATATAT-3’)
ChloR R1 (5’-TTACGCCCCGCCCTGCCACTCATCGCAGTA-3’)

The coding regions were ligated into the yeast expression vector pYES2.1 using a TOPO ligation kit (#K415001) (Thermo Fisher Scientific, Waltham, Massachusetts, US).

Yeast cells were grown in Yeast Peptone Dextrose medium (#Y1375) (Sigma-Aldrich, St. Louis, Missouri, US) or Yeast Nitrogenous Base medium (#233520) (BD, Franklin Lakes, New Jersey, US) with 2% glucose for culture propagation at 30 °C. Experimental cultures were grown in YNB medium enriched with 2% galactose with various additives such as uracil (final concentration 20 μg/ml) or chloramphenicol (final concentration 4 mg/ml) depending on the experiment. Yeast growth curve standards were made using a haemocytometer and measuring the corresponding absorbance value at OD600 in a 1 cm cuvette using a Nanodrop 200c spectrophotometer (Thermo Fisher Scientific, Waltham, Massachusetts, US).

### Construction of transformation plasmids

Yeast expression plasmids for the chloramphenicol resistance gene function test were synthesized using the pYES2.1 TOPO TA Yeast Expression Kit. The ChloR gene sequence was amplified from the pCR2.1-p35S-ChloR-NosT plasmid while the eGFP gene sequence (which was used as a negative control) was amplified from the pSpCas9n(BB)-2A-GFP (PX461) plasmid from Addgene (http://www.addgene.org/48140/) (Table 4).

**Table 4:** List of primers used to construct expression cassettes for *Symbiodinium* expression vectors.

Used in plasmid	Name	Function	Sequence	Length
p35S-ChloR-NosT	p35S F1	Amplify p35S	CTAGAGCCAAGCTGATCTCCT	21
p35S-ChloR-NosT	p35S-CAT R1	Amplify p35S	GGTGGTATATCCAGTGATTTTTTTCTCCATACCGGTCGGAGTCCTCTCCAAATGAAATGA	60
p35S-ChloR-NosT	p35S-CAT F2	Amplify ChloR	ATGGAGAAAAAAATCACTGGATAT	24
p35S-ChloR-NosT	CAT-NosT R2	Amplify ChloR	CCAAATGTTTGAACGATCTGCTTGACAAGCCAATTGTTACGCCCCGCCCTGCCACTCATC	60
p35S-ChloR-NosT	CAT-NosT F3	Amplify NosT	GATGAGTGGCAGGGCGGGGCGTAACAATTGGCTTGTCAAGCAGATCGTTCAAACATTTGG	60
p35S-ChloR-NosT	NosT R3	Amplify NosT	TCGATCTAGTAACATAGATG	20
pAct-ChloR-ActT	pActA F1	Amplify pActA	GGAGACCCGGAAACGTGTCG	20
pAct-ChloR-ActT	pActA-CAT R1	Amplify pActA	CCATGTTTAAACACCTTAAGGTCGGGCTAACTCCGAGACACTTCGTGGAA	50
pAct-ChloR-ActT	pActA-CAT F2	Amplify ChloR	TTAGCCCGACCTTAAGGTGTTTAAACATGGAGAAAAAAATCACTGGATAT	50
pAct-ChloR-ActT	CAT-ActT R2	Amplify ChloR	AGACCGTGGGGCGTCGACACGCGTTTACGCCCCGCCCTGCCACTC	45
pAct-ChloR-ActT	CAT-ActT F3	Amplify ActT	CGGGGCGTAAACGCGTGTCGACGCCCCACGGTCTCATTTTGGACTAA	47
pAct-ChloR-ActT	ActT R3	Amplify ActT	ACTACAAGGGCGTTTTTTTTTTTCAGTG	28
pBTubA-ChloR	pBTubA F1	Amplify pBTubA	CTAAATGTCAAGCAACTCAATAT	23
pBTubA-ChloR	pBTubA-CAT R1	Amplify pBTubA	TCCATGTTTAAACCTTAAGGTCGACGGCTGCAAGAAGCGGAAGGTA	46
pBTubA-ChloR	CAT F1	Amplify ChloR	GTCGACCTTAAGGTTTAAACATGGAGAAAAAAATCACTGGATATACCACC	50
pBTubA-ChloR	Chlo R3	Amplify ChloR	TTACGCCCCGCCCTGCCACTCATCGCAGTA	30
pBTubB-ChloR	pBTubB F1	Amplify pBTubB	ACATGGTCATAGACGTTACCTAGCT	25
pBTubB-ChloR	pBTubB-CAT R1	Amplify pBTubB	TCTCCATGTTTAAACCTTAAGGTCGACGGCGGAAGGCTGCAGAAAAAGGA	50
pBTubB-ChloR	CAT F1	Amplify ChloR	GTCGACCTTAAGGTTTAAACATGGAGAAAAAAATCACTGGATATACCACC	50
pBTubB-ChloR	Chlo R3	Amplify ChloR	TTACGCCCCGCCCTGCCACTCATCGCAGTA	30
pHsp90-ChloR-Hsp90T	pHsp90 F1	Amplify pHsp90	GTCGACCTTGCATAGGGGAATCGTACTTCCAA	32
pHsp90-ChloR-Hsp90T	pHsp90-ChloR R1	Amplify pHsp90	CATGTTTAAACCTTAAGGTCGACGGCTCTCTGGACACGCAAGTTTC	46
pHsp90-ChloR-Hsp90T	pHsp90-ChloR F2	Amplify ChloR	GCCGTCGACCTTAAGGTTTAAACATGGAGAAAAAAATCACTGGATATACCAC	52
pHsp90-ChloR-Hsp90T	ChloR-Hsp90T R2	Amplify ChloR	AGCGCTAGCCTTAAGGTTTAAACTTACGCCCCGCCCTGCCA	41
pHsp90-ChloR-Hsp90T	ChloR-Hsp90T F3	Amplify Hsp90T	TAAGTTTAAACCTTAAGGCTAGCGCTGTTTGATGACGGGTGCAGG	45
pHsp90-ChloR-Hsp90T	Hsp90T R3	Amplify Hsp90T	GCTAGCAGGACCCCTGGTCAGTCCCGTT	28
pPsbJ-ChloR	pPsbJ F1	Amplify pPsbJ	GTCGACCTGTCAGCAGCCATTTTGATCACATT	32
pPsbJ-ChloR	pPsbJ-ChloR R1	Amplify pPsbJ	TCCATGTTTAAACCTTAAGGTCGACGCTCACTTGCACCGACCGGCC	46
pPsbJ-ChloR	pPsbJ-ChloR F2	Amplify ChloR	TGAGCGTCGACCTTAAGGTTTAAACATGGAGAAAAAAATCACTGGATATACCA	53
pPsbJ-ChloR	Chlo R3	Amplify ChloR	TTACGCCCCGCCCTGCCACTCATCGCAGTA	30
pYESChloR	Chlo F3	Clone ChloR	ATGGAGAAAAAAATCACTGGATATACCACCGTTGATATAT	40
pYESChloR	Chlo R3	Clone ChloR	TTACGCCCCGCCCTGCCACTCATCGCAGTA	30
pYESeGFP	eGFP clone F1	Clone eGFP	TCGACCTGCAGGCGGCCGCAC	21
pYESeGFP	eGFP R1	Clone eGFP	CTTCTTGTCCATGGTGAAGGGG	22
pPsbAGEM	AIKconstruct2_F	Clone minicircle	GGTCTGGACTTAGTTTATAAGGC	23
pPsbAGEM	AIKconstruct2_R	Clone minicircle	AGACAAGTCTGGTCTGGACG	22
pPsbA^S264G^GEM	AIKcon2mut1	psbA mutagenesis	GAAGAAGTGTAAACTACGAGAGTTATTGAAGCCAGCATATTGAAAAATTAGTCTGCCAAA	60
pPsbA^S264G^GEM	AIKcon2mut2	psbA mutagenesis	TTTGGCAGACTAATTTTTCAATATGCTGGCTTCAATAACTCTCGTAGTTTACACTTCTTC	60

pCR2.1-based plasmids were synthesized using the pCR2.1 plasmid from the TOPO TA cloning kit as the vector backbone. The p35S-ChloR-NosT cassette was constructed by amplifying the p35S promoter and the NosT terminator from the pK7WGF2::hCas9 plasmid from Addgene (http://www.addgene.org/46965/) and amplifying the chloramphenicol acetyltransferase gene from a pBC SK+ plasmid. The three fragments were then assembled using assembly PCR to create a p35S-ChloR-NosT fragment that was subsequently ligated into a pCR2.1 plasmid. Similar methods were used to construct the various other transformation constructs, although in some cases, Gibson assembly rather than normal assembly PCR was used. Primers used for these assembly PCRs can be found in Table 4.

PCR for amplification of the *psbA* minicircle was carried out using MasterAmp PCR buffer D (Epicentre), GoTaq DNA polymerase and primers AIKconstruct2_F and AIKconstruct2_R (Table 4). PCR cycling conditions were 95 °C 2 minutes 15 seconds, followed by 40 cycles of 95°C 45 seconds, 57 °C 45 seconds, 72 °C 3 minutes 30 seconds, followed by a final step of 72 °C 10 minutes. Purified PCR products of the desired size were there ligated using the pGEM-T-Easy Vector System (Promega, Madison, Wisconsin, US). Correct insertions were selected from the subsequently transformed *E*. *coli* clones.

Primers for mutagenesis were designed using the QuikChange Primer Design program. The program designed a pair of mutagenic primers whose sequences are indicated in Table 4. Mutagenesis reactions used 125 ng of each primer, 200 μM each NTP, MasterAmp PCR buffer D (Epicentre) and 2.5 U PfuUltra HF DNA polymerase in a total volume of 50 μl. DNA template concentration was varied and either 5, 10, 20 or 50 ng of pPsbAGEM DNA was used per reaction. PCR cycling conditions were 95 °C 30 seconds, followed by 16 cycles of 95 °C 30 seconds, 55 °C 1 minute, 72 °C 8 minutes, followed by a final step of 72 °C 10 minutes. Samples were subsequently treated with 20 U *Dpn*I restriction enzyme for 1 hour at 37 °C. 5μl of reaction mix was used to transform chemically competent DH5α cells. Cells were plated out on LB agar plates containing 100 μg/ml ampicillin. Resulting colonies were picked and cultured. Sequencing of plasmid DNA from these cells revealed the presence of the desired base changes from the original construct and no other changes.

pChlamy_3-based plasmids conferring geneticin resistance was kindly provided by Dr. Rachel A. Levin from the School of Biological Earth and Environmental Sciences, University of New South Wales and Prof. Madeleine van Oppen from the School of BioSciences, University of Melbourne.

Vector map image in Fig 5 was made using PlasMapper [25]. Plasmid maps in the Supporting Information can be viewed using the free plasmid map viewing tool, ApE (http://biologylabs.utah.edu/jorgensen/wayned/ape/).

### *Symbiodinium* cell culture conditions

Cultures of the dinoflagellate *Symbiodinium microadriaticum* (strain CCMP2467) were obtained from the Bigelow National Center for Marine Algae and Microbiota (NCMA). This strain was originally isolated from a scleractinian coral, *Stylophora pistillata*, in the Gulf of Aqaba [26, 27]. Stock cultures were grown in Percival incubators under a 12:12 day:night regiment in f/2 medium in Nunc cell culture-treated TripleFlasks (132913) (ThermoFisher Scientific, Waltham, MA, USA) without shaking. Growth conditions were set at a Photosynthetic Photon Flux Density of 80 μmol photons m^-2^ s^-1^, growth temperature of 26 °C and growth media salinity of 40 ppt (i.e. the salinity of seawater from the Red Sea). These growth conditions will be referred to henceforth as standard culture conditions.

Cultures of the dinoflagellate *Symbiodinium microadriaticum* (strain CS-153) were obtained from the Australian National Algae Culture Collection (ANACC) in Hobart, Tasmania. This strain was originally isolated from a jellyfish, *Cassiopeia xamachana*, from off the coast of Florida, United States of America (ANACC database). Stock cultures were grown in a New Brunswick Innova 4340 incubator under a 14:10 day:night regiment in f/2 medium in Pyrex conical flasks (Corning Incorporated, Corning, NY, USA) without shaking. Growth conditions were set at a Photosynthetic Photon Flux Density of 40 μmol photons m^-2^ s-^1^, growth temperature of 26 °C and growth medium salinity of 34 ppt using Ultramarine synthetic sea salt (Waterlife Research Industries Ltd, Waterlife Research Industries Ltd, Middlesex, United Kingdom).

### Cell counting methods

*Symbiodinium* cell densities were measured using a FlowCAM Nano (Fluid Imaging Technologies Inc, Scarborough, Maine, USA) using manufacturer instructions. *S*. *cerevisiae* cell densities were estimated initially using manual Haemocytometer counting to establish a standard curve of cell density vs OD_600_. The standard curve was then used to estimate all subsequent experimental cell densities that were measured using only OD_600_ values using a Nanodrop 2000c (Thermo Fisher Scientific, Waltham, Massachusetts, USA).

### Transformation methods

#### Silicon carbide whiskers agitation transformation

This protocol was modified from Ten Lohuis and Miller (1998). Approximately 5 × 10^7^ cells were harvested by centrifugation at 3000*g* for 5 minutes, washed with 5 ml of f/2 medium, repelleted, and then resuspended in 500 μl of f/2 medium. A transformation mixture containing 40 μl of 50 μg/ml Silar silicon carbide whiskers (997002-5g) (Haydale Technologies Incorporated, Greer, South Carolina, USA) was sequentially mixed with 20 to 40 μg of plasmid (circular or linear), 160 μl of PEG8000 (20% wv; filter sterilized) and then f/2 medium was added to a final volume of 250 μl. For negative DNA controls, plasmids were omitted from the transformation mix.

The 500 μl resuspended cells were then added to the transformation mixture and vortexed over a period of 2 minutes, pausing for 5 seconds every 10 seconds. 2 ml of f/2 medium with 100 μg/ml of carbenicillin was added to the transformation mixture. The mixture was then incubated in the dark at 27 °C for 1 to 2 days. The agitated *Symbiodinium* were then grown under selection in either liquid culture (1 ml of transformant culture in 150 ml of liquid f/2 medium) or on agar plates (150 μl of transformant culture per agar plate).

#### Biolistics

The biolistics protocol was modified from instructions given in the Biorad PDS-1000/He Biolistic Particle Delivery System manual. Approximately 5 × 10^7^ Symbiodinium cells were harvested by centrifugation at 3000*g* for 5 minutes and subsequently washed with 5 ml of filtered, sterilized f/2 medium before being plated onto an f/2 agar plate approximately 1 hour before transformation. Tungsten or gold microcarriers suspended in 50% glycerol (30 mg/ml) were vortexed for 5 minutes before a 50 μl aliquot of the mix was placed into a 1.5 ml Eppendorf tube. 5 μg of plasmid DNA was added to the microcarrier mix (omitted for the negative DNA control) and immediately vortexed for 10 seconds. This was followed by the addition of 50 μl of 2.5 M CaCl_2_, vortexing for 10 seconds, and the addition of 20 μl 0.1 M spermidine. The mixture was then mixed by vortexing for 2 minutes before being allowed to settle for 1 minute. The microcarriers were then pelleted by pulse centrifugation and the liquid supernatant was discarded. The pellet was gently washed with 140 μl of 70% ethanol before the mix was pulse centrifuged and the supernatant was removed. This washing step was repeated with 140 μl of 100% ethanol. The pellet was then re-suspended with 10 or 70 μl of 100% ethanol depending on the number of macrocarriers being used. 10 μl of the mix was used to dip dry a layer of microcarriers onto a Bio-Rad biolistics macrocarrier (#1652335) (Bio-Rad, Hercules, California, US) and this step was repeated seven times when using 7 macrocarriers. The Biorad macrocarrier was then loaded into a macrocarrier holder and fired at an agar plate of *Symbiodinium* cells according to the manufacturer’s manual for the Biorad PDS-1000/He Biolistic Particle Delivery System. Chamber pressure, rupture disk pressure and distance of agar plate from the macrocarrier holder were adjusted based on the experiments carried out as described in the Results section.

After the biolistic bombardment, *Symbiodinium* cells were transferred from the agar plate into 5 ml of f/2 medium and incubated under standard culture conditions for one day. The transformed *Symbiodinium* were then grown under selection in liquid culture (2 ml of transformant culture in 150 ml of liquid f/2 medium) and/or on agar plates (150 μl of transformant culture per agar plate).

#### Electroporation

The electroporation protocol used in this publication was modified from published protocols used for *Phaeodactylum tricornutum* and *Nannochloropsis* sp. [28, 29]. Approximately 1 × 10^8^ cells were harvested by centrifugation at 3000*g* for 5 minutes. The cells were subsequently washed with 5 ml of f/2 medium and repelleted, before being washed with 1 ml of 375 mM sorbitol, repelleted, and finally resuspended in 100 μl of 375 mM sorbitol. The suspension was then mixed with 2–4 μg of plasmid DNA (or just 5 μl of water for the no plasmid control) and 40 μg of denatured salmon sperm DNA. The mixture was then incubated on ice for 10 minutes before being transferred into a 2 mm electroporation cuvette. Electroporation was performed using a Bio-Rad Gene Pulser Xcell Electroporation system (Biorad, Hercules, California, US). The parameters of the system (exponential decay vs multiple pulse, field strength, capacitance, shunt resistance) were adjusted according the experiments carried out as described in the Results section.

After electroporation, the cells were immediately transferred to a 15 ml Falcon tube containing 10 ml of f/2 medium with 100 μg/ml carbenicillin and left under standard culture conditions for one day. After this, the cells were then collected by centrifugation at 1500 *g* for 10 minutes and resuspended in 3 ml of f/2 medium. The electroporated *Symbiodinium* were then grown under selection in liquid culture (1 ml of transformant culture in 150 ml of liquid f/2 medium) and/or on agar plates (150 μl of transformant culture per agar plate).

#### Glass bead agitation transformation, with cell wall digest step

The glass bead protocol was modified from Kindle [30] and the cellulase cell wall digest was modified from Levin et al. [31]. Approximately 2 × 10^6^ cells were harvested by centrifugation at 3000*g* for 5 minutes, washed with 5 ml of f/2 medium, repelleted and then resuspended in 1 ml of digestion solution (0.5 M D-sorbitol in filtered autoclaved seawater). Either 0.3 kilounits (KU) of cellulase from *Trichoderma* sp. (#C1794) (Sigma-Aldrich, St. Louis, Missouri, US) or 10 mg of Snailase (S0100, Beijing Biodee Biotechnology Co., Ltd, Beijing, China) was added to the digest mix. The mixture was then incubated in the dark at 30 °C for 1 day on a tube rotator.

The digest mixture was then centrifuged at 800*g* for 5 minutes and the supernatant was discarded. The pellet was resuspended in 1 ml of protoplast wash solution (0.5 M D-sorbitol, 0.5 M sucrose, 25 mM CaCl_2_, and 100 mg/ml kanamycin in filtered autoclaved seawater) before being incubated at 25 °C for 3 hours in the dark on a tube rotator. The cells were centrifuged at 800*g* for 5 minutes before being washed with 1 ml of f/2 medium, repelleted, and resuspended in 300 μl of f/2 medium.

100 μl of filter sterilized 20% w/v PEG 6000 (81260, Sigma) was added to the 300 μl of resuspended cells followed by the addition of 50 μg of salmon sperm carrier DNA (#15632011) (Invitrogen, Carlsbad, California, US) and 2 μg of plasmid DNA (in various combinations of circular and linearized plasmids, as described in the results section). 300 μg of autoclave-sterilized 0.5mm glass beads (#11079105) (BioSpec, Bartlesville, Oklahoma, US) were added to the transformation mixture. The mixture was vortexed for 30 seconds after which the beads were allowed to settle. The agitated *Symbiodinium* were then resuspended in 2 ml of fresh f/2 with 100 μg/ml of carbenicillin. After this, the transformant cultures were grown under selection in liquid medium (1 ml of transformant culture in 150 ml of liquid f/2 medium) and on agar plates plates (150 μl of transformant culture per agar plate).

#### FuGENE HD transfection

Approximately 5 × 10^6^ *Symbiodinium* cells were harvested by centrifugation at 3000*g* for 5 minutes, washed with 5ml of f/2 medium, repelleted and resuspended in 150 μl of f/2 medium. A 30 μl FuGENE transfection/DNA mixture was made using f/2 medium with different mixtures of plasmid DNA and FuGENE HD transfection reagent as according to S4 Table. 1 to 30 μl of this mix was then added to the 150 μl cell suspension and incubated at standard growth conditions for 24 hours. The cells were then grown under selection in liquid culture (100 μl of transformant culture per 150 ml of liquid f/2 medium).

#### f/2 agar plate selection

1.5% microbiological agar (Fisher Scientific, New Hampshire, US) or 0.8% plant agar (Duchefa Biochemie, Haarlem, The Netherlands) made up with filtered, autoclaved seawater enriched with f/2 medium was used to make agar plates for transformation selection. 100 μg/ml chloramphenicol or 200 to 1000 ng/ml atrazine was used as the selection antibiotic/herbicide depending on the construct being tested. Control plates with no selection antibiotic/herbicide were also made to confirm that the transformation method was mild enough that some cells survived the treatment. The samples were inoculated onto the agar plate using 3 ml of top agar made from 0.8% plant agar (Duchefa Biochemie, Haarlem, Netherlands) in f/2 medium with no antibiotics. This was done due to the tendency of *Symbiodinium* cells to clump, which prevented the cells from being easily evenly spread across agar surfaces. The plates were then grown in a LMS vertical incubator set to 26 °C with a 14:10 hours day:night cycle and 40 μmol photons m^-2^ s^-1^ light intensity for up to four months.

#### Liquid f/2 selection

Transformant cultures were grown in 225 cm^2^ EasYFlask Nunc bottles (#159934) (Thermo Scientific, Waltham, Massachusetts, US) filled with 150 ml of filtered, autoclaved seawater enriched with f/2 medium. The selection antibiotic/herbicide used was either 100 μg/ml chloramphenicol or 150 ng/ml atrazine and the cultures were kept under standard growth conditions.

## Supporting information

S1 Fig Verification of pPsbJ 5’ UTR sequence via RACE. (PPTX)

S1 Table Price of antibiotics from various major suppliers, taken on 30^th^ of April 2017. (XLSX)

S2 Table Growth curves of Symbiodinium microadriaticum under atrazine treatment. (XLSX)

S3 Table Expression of selected genes from transcriptomics analysis published in Chen, Cui [11]. (XLSX)

S4 Table Detailed list of transformations carried out, with information on transformation conditions and materials used. (XLSX)

S1 File Vector sequence for plasmids pYESChloR. (APE)

S2 File Vector sequence for plasmid pYESeGFP. (APE)

S3 File Genomic sequence of putative β-tubulin A. Based on sequence from Smic.scaffold612 published in Aranda, Li [10].
(STR)

S4 File Genomic sequence of putative β-tubulin B. Based on sequence from Smic.scaffold51 published in Aranda, Li [10].
(STR)

S5 File Genomic sequence of putative Hsp90. Based on sequence from Smic.scaffold975 published in Aranda, Li [10].
(STR)

S6 File Vector sequence for plasmid pCR4 p35S-ChloR-NosT. (APE)

S7 File Vector sequence for plasmid pCR2.1 pAct-ChloR-ActT. (APE)

S8 File Vector sequence for plasmid pCR2.1 pBtubA-ChloR. (STR)

S9 File Vector sequence for plasmid pCR2.1 pBtubB-ChloR. (STR)

S10 File Vector sequence for plasmid pCR2.1 pHsp90-ChloR-Hsp90T. (STR)

S11 File Vector sequence for plasmid pCR2.1 pPsbJ-ChloR. (APE)

S12 File Vector sequence for plasmid pPsbA^S264G^GEM. Atrazine resistance is predicted to be conferred by mutation of nucleotide 790 T->G and 791 C->G, causing a 264 Ser->Gly mutation. A silent mutation for identification purposes was also added to 795 T->C.
(APE)

S13 File Vector sequence for plasmid pPsbAGEM. (APE)

S14 File Vector sequence for plasmid pChlamy3-GenR-GAmCherry. (APE)

S15 File Vector sequence for plasmid p35S-GenR-eCFP-NosT. (APE)

S16 File Raw cell count values of *Symbiodinium* cells growing under various antibiotic treatments as measured from using a FlowCAM. These values were used to make the graph shown in Fig 1.
(XLSX)

S17 File Raw OD600 absorbance values of *S*. *cerevisiae* cells growing under chloramphenicol and/or uracil depletion/enrichment. These values were used to make the graph shown in Fig 2.
(XLSX)

## References

[1] Muscatine L, Mccloskey LR, Marian RE. Estimating the daily contribution of carbon from zooxanthellae to coral animal respiration. Limnology and Oceanography. 1981;26(4):601–11.

[2] Muscatine L, Porter JW. Reef corals—mutualistic symbioses adapted to nutrient-poor environments. Bioscience. 1977;27(7):454–60. 10.2307/1297526

[3] Brown BE. Coral bleaching: causes and consequences. Coral Reefs. 1997;16:S129–S38. 10.1007/s003380050249

[4] Hughes TP, Kerry JT, Alvarez-Noriega M, Alvarez-Romero JG, Anderson KD, Baird AH, et al Global warming and recurrent mass bleaching of corals. Nature. 2017;543(7645):373–7. 10.1038/nature21707 . 28300113

[5] Smith DM, Cusack S, Colman AW, Folland CK, Harris GR, Murphy JM. Improved surface temperature prediction for the coming decade from a global climate model. Science. 2007;317(5839):796–9. 10.1126/science.1139540 17690292

[6] Howells EJ, Beltran VH, Larsen NW, Bay LK, Willis BL, van Oppen MJH. Coral thermal tolerance shaped by local adaptation of photosymbionts. Nat Clim Change. 2012;2(2):116–20. 10.1038/Nclimate1330

[7] ten Lohuis MR, Miller DJ. Genetic transformation of dinoflagellates (Amphidinium and Symbiodinium): expression of GUS in microalgae using heterologous promoter constructs. Plant J. 1998;13(3):427–35.

[8] Ortiz-Matamoros MF, Islas-Flores T, Voigt B, Menzel D, Baluska F, Villanueva MA. Heterologous DNA Uptake in Cultured Symbiodinium spp. Aided by Agrobacterium tumefaciens. PLoS One. 2015;10(7):e0132693 10.1371/journal.pone.0132693 . 26167858 PMC4500500

[9] Ortiz-Matamoros MF, Villanueva MA, Islas-Flores T. Transient transformation of cultured photosynthetic dinoflagellates (Symbiodinium spp.) with plant-targeted vectors. Cienc 3 2015;41(1):21–32. 10.7773/cm.v41i1.2449

[10] Aranda M, Li Y, Liew YJ, Baumgarten S, Simakov O, Wilson MC, et al Genomes of coral dinoflagellate symbionts highlight evolutionary adaptations conducive to a symbiotic lifestyle. Scientific Reports. 2016;6:39734 ARTN 39734 10.1038/srep39734 28004835 PMC5177918

[11] Chen JE, Cui G, Wang X, Liew YJ, Aranda M. Recent expansion of heat-activated retrotransposons in the coral symbiont Symbiodinium microadriaticum. ISME J. 2017;12: 639–43. Epub 2017/10/21. 10.1038/ismej.2017.179 . 29053149 PMC5776459

[12] Zhang H, Zhuang YY, Gill J, Lin SJ. Proof that Dinoflagellate Spliced Leader (DinoSL) is a Useful Hook for Fishing Dinoflagellate Transcripts from Mixed Microbial Samples: Symbiodinium kawagutii as a Case Study. Protist. 2013;164(4):510–27. 10.1016/j.protis.2013.04.002 23773861

[13] Lin SJ, Cheng SF, Song B, Zhong X, Lin X, Li WJ, et al The Symbiodinium kawagutii genome illuminates dinoflagellate gene expression and coral symbiosis. Science. 2015;350(6261):691–4. 10.1126/science.aad0408 26542574

[14] Howe CJ, Nisbet RE, Barbrook AC. The remarkable chloroplast genome of dinoflagellates. J Exp Bot. 2008;59(5):1035–45. 10.1093/jxb/erm292 . 18319241

[15] Gornik SG, Ford KL, Mulhern TD, Bacic A, McFadden GI, Waller RFJCB. Loss of nucleosomal DNA condensation coincides with appearance of a novel nuclear protein in dinoflagellates. 2012;22(24):2303–12. 10.1016/j.cub.2012.10.036 23159597

[16] Herzog M, Von Boletzky S, Soyer M-OJOol. Ultrastructural and biochemical nuclear aspects of eukaryote classification: independent evolution of the dinoflagellates as a sister group of the actual eukaryotes? 1984;13(3–4):205–15.

[17] Nimmo I, Barbrook AC, Chen JE, Geisler K, Smith AG, Aranda M, et al Genetic transformation of the dinoflagellate chloroplast. 2018. 10.7554/eLife.45292 PMC6639071 31317866

[18] Blank RJ, Huss VA, Kersten WJAom. Base composition of DNA from symbiotic dinoflagellates: a tool for phylogenetic classification. 1988;149(6):515–20.

[19] Krieg AM, Yi AK, Matson S, Waldschmidt TJ, Bishop GA, Teasdale R, et al CpG motifs in bacterial DNA trigger direct B-cell activation. Nature. 1995;374(6522):546–9. Epub 1995/04/06. 10.1038/374546a0 . 7700380

[20] Razin A. CpG methylation, chromatin structure and gene silencing-a three-way connection. EMBO J. 1998;17(17):4905–8. Epub 1998/09/02. 10.1093/emboj/17.17.4905 . 9724627 PMC1170819

[21] Pfaffeneder T, Spada F, Wagner M, Brandmayr C, Laube SK, Eisen D, et al Tet oxidizes thymine to 5-hydroxymethyluracil in mouse embryonic stem cell DNA. Nat Chem Biol. 2014;10(7):574–81. Epub 2014/05/20. 10.1038/nchembio.1532 . 24838012

[22] Rae PM. 5-Hydroxymethyluracil in the DNA of a dinoflagellate. Proc Natl Acad Sci U S A. 1973;70(4):1141–5. Epub 1973/04/01. . 4515611 10.1073/pnas.70.4.1141 PMC433443

[23] Kawasaki F, Beraldi D, Hardisty RE, McInroy GR, van Delft P, Balasubramanian S. Genome-wide mapping of 5-hydroxymethyluracil in the eukaryote parasite Leishmania. Genome Biol. 2017;18(1):23 Epub 2017/02/01. 10.1186/s13059-017-1150-1 . 28137275 PMC5282726

[24] Yu M, Hon GC, Szulwach KE, Song CX, Jin P, Ren B, et al Tet-assisted bisulfite sequencing of 5-hydroxymethylcytosine. Nat Protoc. 2012;7(12):2159–70. Epub 2012/12/01. 10.1038/nprot.2012.137 . 23196972 PMC3641661

[25] Dong X, Stothard P, Forsythe IJ, Wishart DS. PlasMapper: a web server for drawing and auto-annotating plasmid maps. Nucleic Acids Res. 2004;32(Web Server issue):W660–4. Epub 2004/06/25. 10.1093/nar/gkh410 . 15215471 PMC441548

[26] LaJeunesse TC. Investigating the biodiversity, ecology, and phylogeny of endosymbiotic dinoflagellates in the genus Symbiodinium using the its region: In search of a "species" level marker. J Phycol. 2001;37(5):866–80. 10.1046/j.1529-8817.2001.01031.x

[27] Lajeunesse TC, Lee SY, Gil-Agudelo DL, Knowlton N, Jeong HJ. Symbiodinium necroappetens sp nov (Dinophyceae): an opportunist ‘zooxanthella’ found in bleached and diseased tissues of Caribbean reef corals. Eur J Phycol. 2015;50(2):223–38. 10.1080/09670262.2015.1025857

[28] Kilian O, Benemann CSE, Niyogi KK, Vick B. High-efficiency homologous recombination in the oil-producing alga Nannochloropsis sp. P Natl Acad Sci USA. 2011;108(52):21265–9. 10.1073/pnas.1105861108 22123974 PMC3248512

[29] Zhang CY, Hu HH. High-efficiency nuclear transformation of the diatom Phaeodactylum tricornutum by electroporation. Mar Genom. 2014;16:63–6. 10.1016/j.margen.2013.10.003 24269346

[30] Kindle KL. High-frequency nuclear transformation of Chlamydomonas reinhardtii. P Natl Acad Sci USA. 1990;87(3):1228–32. . 2105499 10.1073/pnas.87.3.1228 PMC53444

[31] Levin RA, Suggett DJ, Nitschke MR, van Oppen MJ, Steinberg PD. Expanding the Symbiodinium (Dinophyceae, Suessiales) Toolkit Through Protoplast Technology. J Eukaryot Microbiol. 2017; 64(5):588–97. 10.1111/jeu.12393 . 28120360

