# Supplementary material for: The genetic intractability of Symbiodinium microadriaticum to standard algal transformation methods
Source: PLoS One. 2019 Feb 19;14(2):e0211936. doi: 10.1371/journal.pone.0211936 (PMC6380556; doi:10.1371/journal.pone.0211936)
Supplement: S1 Fig — (PPTX) [file pone.0211936.s001.pptx]

## Slide 1
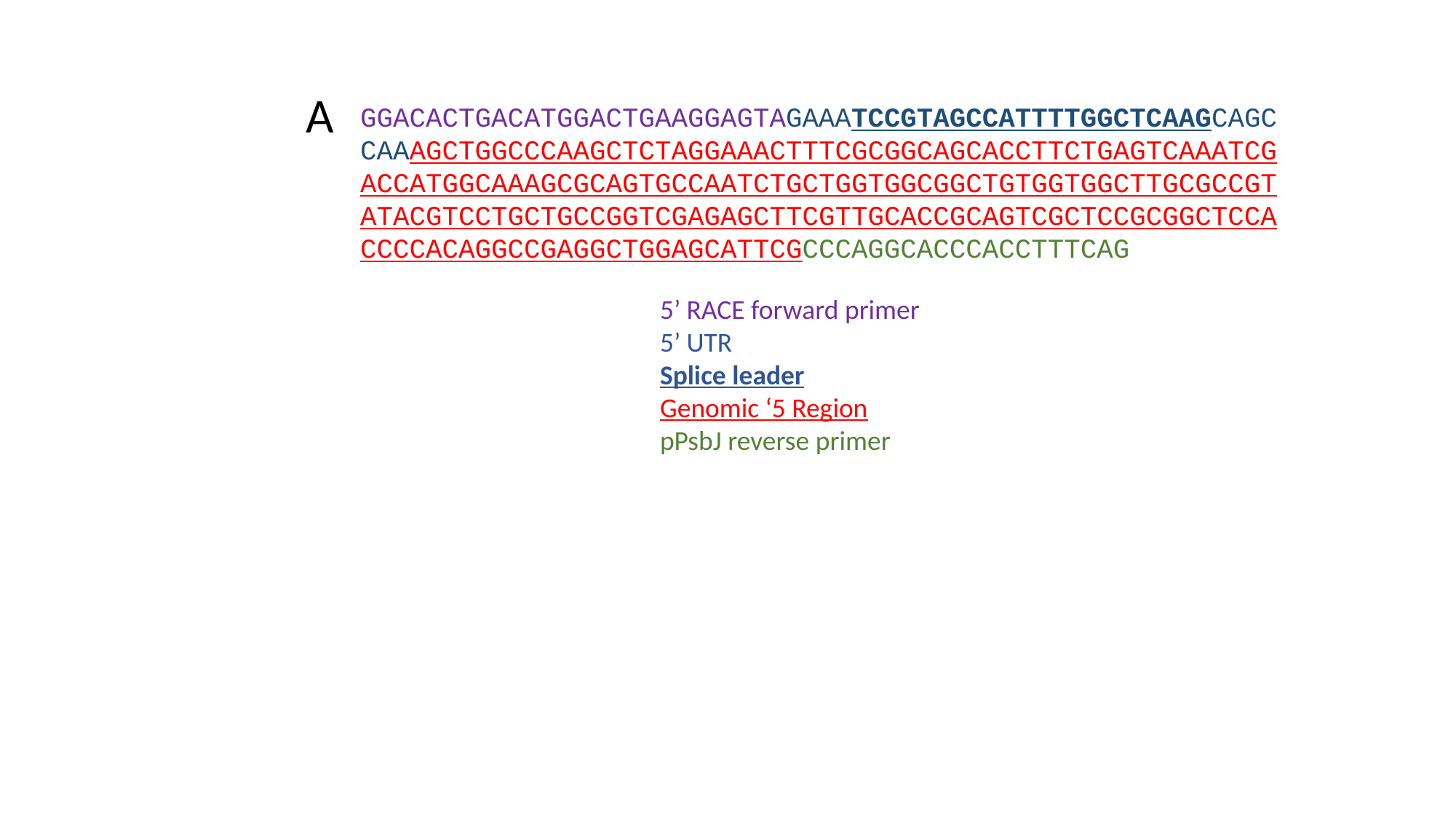

A
GGACACTGACATGGACTGAAGGAGTAGAAATCCGTAGCCATTTTGGCTCAAGCAGCCAAAGCTGGCCCAAGCTCTAGGAAACTTTCGCGGCAGCACCTTCTGAGTCAAATCGACCATGGCAAAGCGCAGTGCCAATCTGCTGGTGGCGGCTGTGGTGGCTTGCGCCGTATACGTCCTGCTGCCGGTCGAGAGCTTCGTTGCACCGCAGTCGCTCCGCGGCTCCACCCCACAGGCCGAGGCTGGAGCATTCGCCCAGGCACCCACCTTTCAG
5’ RACE forward primer
5’ UTR
Splice leader
Genomic ‘5 Region
pPsbJ reverse primer

## Slide 2
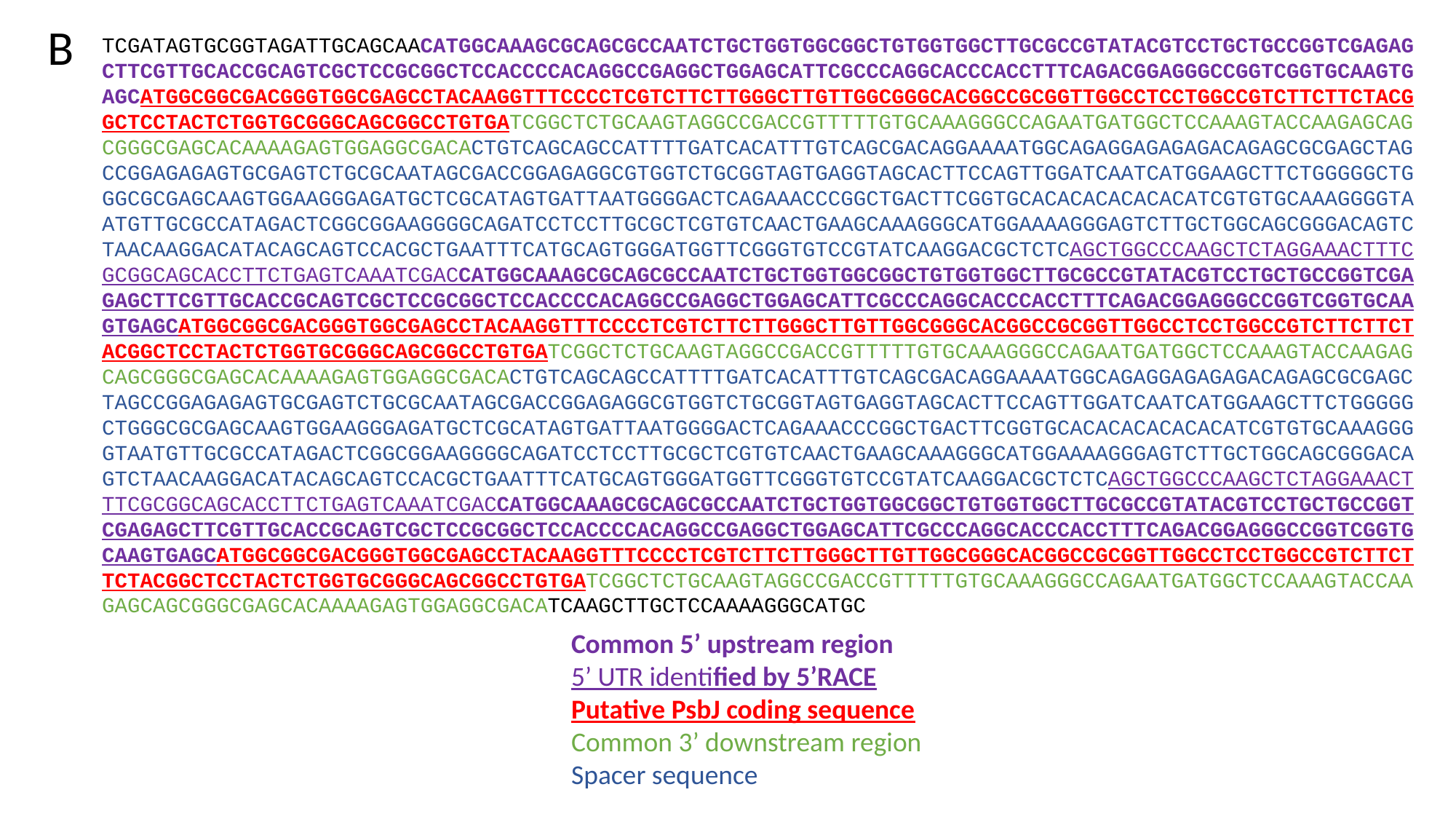

B
TCGATAGTGCGGTAGATTGCAGCAACATGGCAAAGCGCAGCGCCAATCTGCTGGTGGCGGCTGTGGTGGCTTGCGCCGTATACGTCCTGCTGCCGGTCGAGAGCTTCGTTGCACCGCAGTCGCTCCGCGGCTCCACCCCACAGGCCGAGGCTGGAGCATTCGCCCAGGCACCCACCTTTCAGACGGAGGGCCGGTCGGTGCAAGTGAGCATGGCGGCGACGGGTGGCGAGCCTACAAGGTTTCCCCTCGTCTTCTTGGGCTTGTTGGCGGGCACGGCCGCGGTTGGCCTCCTGGCCGTCTTCTTCTACGGCTCCTACTCTGGTGCGGGCAGCGGCCTGTGATCGGCTCTGCAAGTAGGCCGACCGTTTTTGTGCAAAGGGCCAGAATGATGGCTCCAAAGTACCAAGAGCAGCGGGCGAGCACAAAAGAGTGGAGGCGACACTGTCAGCAGCCATTTTGATCACATTTGTCAGCGACAGGAAAATGGCAGAGGAGAGAGACAGAGCGCGAGCTAGCCGGAGAGAGTGCGAGTCTGCGCAATAGCGACCGGAGAGGCGTGGTCTGCGGTAGTGAGGTAGCACTTCCAGTTGGATCAATCATGGAAGCTTCTGGGGGCTGGGCGCGAGCAAGTGGAAGGGAGATGCTCGCATAGTGATTAATGGGGACTCAGAAACCCGGCTGACTTCGGTGCACACACACACACATCGTGTGCAAAGGGGTAATGTTGCGCCATAGACTCGGCGGAAGGGGCAGATCCTCCTTGCGCTCGTGTCAACTGAAGCAAAGGGCATGGAAAAGGGAGTCTTGCTGGCAGCGGGACAGTCTAACAAGGACATACAGCAGTCCACGCTGAATTTCATGCAGTGGGATGGTTCGGGTGTCCGTATCAAGGACGCTCTCAGCTGGCCCAAGCTCTAGGAAACTTTCGCGGCAGCACCTTCTGAGTCAAATCGACCATGGCAAAGCGCAGCGCCAATCTGCTGGTGGCGGCTGTGGTGGCTTGCGCCGTATACGTCCTGCTGCCGGTCGAGAGCTTCGTTGCACCGCAGTCGCTCCGCGGCTCCACCCCACAGGCCGAGGCTGGAGCATTCGCCCAGGCACCCACCTTTCAGACGGAGGGCCGGTCGGTGCAAGTGAGCATGGCGGCGACGGGTGGCGAGCCTACAAGGTTTCCCCTCGTCTTCTTGGGCTTGTTGGCGGGCACGGCCGCGGTTGGCCTCCTGGCCGTCTTCTTCTACGGCTCCTACTCTGGTGCGGGCAGCGGCCTGTGATCGGCTCTGCAAGTAGGCCGACCGTTTTTGTGCAAAGGGCCAGAATGATGGCTCCAAAGTACCAAGAGCAGCGGGCGAGCACAAAAGAGTGGAGGCGACACTGTCAGCAGCCATTTTGATCACATTTGTCAGCGACAGGAAAATGGCAGAGGAGAGAGACAGAGCGCGAGCTAGCCGGAGAGAGTGCGAGTCTGCGCAATAGCGACCGGAGAGGCGTGGTCTGCGGTAGTGAGGTAGCACTTCCAGTTGGATCAATCATGGAAGCTTCTGGGGGCTGGGCGCGAGCAAGTGGAAGGGAGATGCTCGCATAGTGATTAATGGGGACTCAGAAACCCGGCTGACTTCGGTGCACACACACACACATCGTGTGCAAAGGGGTAATGTTGCGCCATAGACTCGGCGGAAGGGGCAGATCCTCCTTGCGCTCGTGTCAACTGAAGCAAAGGGCATGGAAAAGGGAGTCTTGCTGGCAGCGGGACAGTCTAACAAGGACATACAGCAGTCCACGCTGAATTTCATGCAGTGGGATGGTTCGGGTGTCCGTATCAAGGACGCTCTCAGCTGGCCCAAGCTCTAGGAAACTTTCGCGGCAGCACCTTCTGAGTCAAATCGACCATGGCAAAGCGCAGCGCCAATCTGCTGGTGGCGGCTGTGGTGGCTTGCGCCGTATACGTCCTGCTGCCGGTCGAGAGCTTCGTTGCACCGCAGTCGCTCCGCGGCTCCACCCCACAGGCCGAGGCTGGAGCATTCGCCCAGGCACCCACCTTTCAGACGGAGGGCCGGTCGGTGCAAGTGAGCATGGCGGCGACGGGTGGCGAGCCTACAAGGTTTCCCCTCGTCTTCTTGGGCTTGTTGGCGGGCACGGCCGCGGTTGGCCTCCTGGCCGTCTTCTTCTACGGCTCCTACTCTGGTGCGGGCAGCGGCCTGTGATCGGCTCTGCAAGTAGGCCGACCGTTTTTGTGCAAAGGGCCAGAATGATGGCTCCAAAGTACCAAGAGCAGCGGGCGAGCACAAAAGAGTGGAGGCGACATCAAGCTTGCTCCAAAAGGGCATGC
Common 5’ upstream region
5’ UTR identified by 5’RACE
Putative PsbJ coding sequence
Common 3’ downstream region
Spacer sequence
